# Examination of self-harm clustering in adolescent peer networks: a nationwide registry cohort study in Finland

**DOI:** 10.1016/j.lanepe.2025.101517

**Published:** 2025-11-02

**Authors:** Jussi Alho, Roger T. Webb, Mai Gutvilig, Ripsa Niemi, Kaisla Komulainen, Kimmo Suokas, Petri Böckerman, Marko Elovainio, Nav Kapur, Christian Hakulinen

**Affiliations:** a Department of Psychology, Faculty of Medicine, University of Helsinki, Helsinki, Finland; b Centre for Mental Health and Safety, Division of Psychology & Mental Health, University of Manchester, Manchester Academic Health Sciences Centre (MAHSC), Manchester, UK; c School of Business and Economics, University of Jyväskylä, Jyväskylä, Finland; d Finnish Institute for Health and Welfare, Helsinki, Finland

**Keywords:** Self-harm, Adolescence, Social transmission, Peer networks, Registry data

## Abstract

**Background:**

Clusters of self-harming behaviour among adolescents have been observed, yet population-based epidemiological evidence is lacking. This study aims to address this lack by examining the clustering of self-harming behaviour within adolescent peer networks at the population level.

**Methods:**

We used nationwide registry data on Finnish people born between January 1, 1985, and December 31, 2000, to examine whether having same-grade schoolmates who had self-harmed was associated with greater subsequent self-harm risk. Cohort members were followed up until first recorded self-harm episode, emigration, death, or December 31, 2020, whichever came first. Hazard ratios (HRs) were estimated using mixed-effects Cox proportional hazards models adjusted for a comprehensive set of individual-, parental-, school-, and area-level covariates.

**Findings:**

The cohort comprised 913,149 Finnish residents. Having same-grade schoolmates who had self-harmed between school-starting age and finishing ninth grade was associated with a higher, albeit small in magnitude, HR of subsequent self-harm over a median of 11.6 years of follow-up (HR 1.05, [95% CI 1.01–1.09]). HR was not consistently higher over follow-up time but was highest in the beginning of follow-up when the cohort members were around age 16 (1.45 [1.25–1.69]). Limiting exposure to schoolmates’ self-harm episodes to 1 year consistently showed the highest risk around age 16, regardless of whether the exposure occurred in ninth grade (1.49 [1.21–1.82]) or eighth grade (1.36 [1.07–1.74]), with follow-up commencing after the respective grade.

**Interpretation:**

While we cannot rule out residual confounding, our findings suggest that self-harm may socially transmit within adolescent peer networks. The observed highest risk around age 16 suggests that external stressors associated with transitioning to new life stages at this age may moderate the impact of peer self-harm exposure. Prevention and intervention measures that consider possible peer influences on adolescents’ self-harming behaviour may help reduce the public health burden of self-harm.

**Funding:**

European Research Council and 10.13039/501100002341Research Council of Finland.

**Research in context**

**Evidence before this study**

We reviewed the literature on the topic by searching for article titles or abstracts in PubMed, EMBASE, and Google Scholar published from inception to October 31, 2024, using the following combination of search terms: “self-harm” or “self-injur” or “self-poison” or “suicid” AND “adolesc” or “youth” or “child” AND “transmi” or “contag” or “spread” or “cluster”. We also reviewed the literature referenced in the identified articles. Previous national registry studies have examined and found strong associations for familial transmission of suicidal behaviour, but no large-scale registry studies were found examining the transmission of self-harming behaviour among adolescent peers. Published studies examining the clustering or transmission of self-harming behaviour in adolescents have been predominantly surveys or case studies.

**Added value of this study**

We conducted a national cohort study of over 900,000 Finnish people from more than 900 lower secondary schools to investigate transmission of self-ham in adolescent peer networks. After accounting for a comprehensive set of covariates at the individual, parental, school, and area levels, we found that having same-grade schoolmates who had self-harmed was associated with a greater subsequent risk of self-harm. The finding that the hazard ratio was highest in the early follow-up period, in the context of institutionally imposed peer networks, suggests that the observed associations are not due to pre-existing risk factors shared by adolescents in the same peer network but could rather result from either social transmission or normalisation of self-harming behaviour among adolescents.

**Implications of all the available evidence**

While some residual confounding is possible, the findings from this observational study suggest that self-harming behaviour may be socially transmitted within adolescent peer networks. More research is needed to better understand the mechanisms underlying the observed associations. Identifying the key peer influences on adolescents’ self-harming behaviour could inform effective prevention and intervention measures, thereby reducing the public health burden of self-harm.

## Introduction

Self-harm, defined as intentional self-poisoning or self-injury with a non-fatal outcome, is a major public health concern among adolescents.^1^^,^^2^ The lifetime prevalence of self-harm in adolescents globally is estimated to be 17–22%.^3^, ^4^, ^5^ Adolescents who have self-harmed also have a greatly elevated suicide risk.^6^, ^7^, ^8^ Evidence from many high-income countries, including Finland, indicates that a growing number of adolescents engaged in self-harming behaviour during recent years.^9^, ^10^, ^11^, ^12^ These concerning trends highlight the contemporary public health importance of developing a greater understanding of adolescent self-harm aetiology.

Risk factors for self-harm in adolescents include childhood abuse, bullying, psychiatric illnesses, substance use, parental divorce, poor family relationships, lack of friends, and the exposure to self-harming behaviour of others.^13^^,^^14^ Self-harm clusters among adolescents have been detected, with direct person-to-person transmission proposed as one possible causal mechanism.^15^ While surveys have found that exposure to peers’ self-harming behaviour is associated with a greater risk of subsequently self-harming among exposed adolescents,^16^, ^17^, ^18^, ^19^, ^20^, ^21^ high-quality population-based registry studies on the social transmission of self-harming behaviour among adolescents are lacking.

The main advantage of using data from population-wide interlinked registers is that they are not susceptible to health-related selection and non-participation biases, which limit most survey-based cohort studies. When analysing social network associations, another potential source of bias stems from assortative relating or homophily,^22^ referring to the tendency of individuals with similar characteristics to socially connect. This self-selection bias can be mitigated by defining institutionally imposed social networks, such as schoolmates, using nationwide registry data.^23^ Due to the considerable time spent together, schoolmates—especially those in the same year or grade—form a significant and mutually influential peer network in adolescence.^24^

In this study, we utilised Finnish nationwide registry data to investigate the association between having same-grade schoolmates in lower secondary school who had self-harmed and subsequent risk of self-harm. We hypothesised a positive relationship between these two variables. Better understanding of potential peer effects in adolescent self-harm could facilitate the development of more effective and targeted prevention and intervention measures, thereby reducing the public health burden of self-harm.

## Methods

### Study population

We included all Finnish residents born between January 1, 1985, and December 31, 2000. The study population's demographic, health, and school information were linked from several national administrative registers using unique identification numbers assigned to all Finnish residents since 1969. Information on schoolmates was obtained from the National Joint Application Register, which contains data for individuals in their final year of lower secondary education (i.e., ninth grade) as they apply for upper secondary education. Individuals with missing school information were excluded. During the study period of 2001–2016, information from 2001 to 2007 was available only for those students who applied for upper secondary education. From 2008 to 2016, information was available for all students at the end of the ninth grade, including those who did not apply for secondary education.

We defined same-grade schoolmates as a proxy for peer networks based on an assumption of high levels of social interaction among them. Although connections within these networks do not reflect actual observed relationships, they provide a meaningful approximation of social exposure. To enhance the stability of the peer networks during secondary school (i.e., schoolmates in the same grade) and to improve the validity of peer network exposure, we excluded individuals who had moved to the school's municipality less than 3 years before ninth grade ended and those who had immigrated to Finland after school starting age (August 1 of the year when they reached age seven). Those who died or emigrated between applying to upper secondary education and the beginning of the follow-up were also excluded. All ninth-grade peer networks with 10 or more remaining cohort members were included in the analyses. For more information on the structure of the Finnish educational systems, see Supplementary Methods S1.

**Fig. 1:**
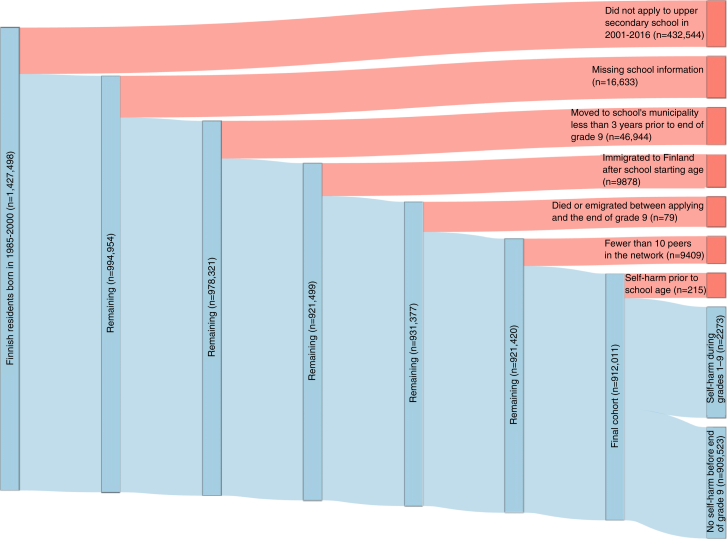
Flowchart (Sankey diagram) of the study population derivation.

Among the total of 912,011 cohort members (median age at the end of ninth grade, 16.0 [IQR, 15.7–16.2] years), 2488 had a self-harm episode (International Statistical Classification of Diseases Tenth Revision [ICD-10] codes X60–X84, Y10–Y34, Y87.0, Y87.2, Z72.8, Z91.5) recorded before the end of ninth grade and were therefore excluded from follow-up. Of these, 2273 had their first self-harm episode recorded after school-starting age (i.e., after August 1 of the year they turned seven) and were used as the main exposure and therefore excluded from the follow-up. The remaining 909,523 cohort members from 909 schools and 11,378 ninth grades were followed from June 1 of the year when they completed ninth grade (around age 16) until the first recorded self-harm episode, death, emigration, or end of follow-up on December 31, 2020, whichever occurred first. The maximum follow-up duration was thus from June 1, 2001, to December 31, 2020. Since the focus of the present study was on non-fatal self-harm, deaths by suicide (n = 57) during the follow-up were not classified as self-harm outcomes. A total of 22,842 cohort members (2.5%) had at least one full sibling or twin in the same school year at the same school, representing 11,243 families. Additionally, 92 cohort members had at least one half-sibling in the same school year at the same school. Given that most siblings in our data (889,077, 97.5%) were of different ages—and therefore were not in the same school grade—we chose not to include family as a random effect in the analyses. The derivation of the study population is illustrated in Fig. 1.

### Self-harming behaviour

Information on self-harming behaviour was acquired from the healthcare registers of the Finnish Institute for Health and Welfare. They contain information on all the inpatient hospital admissions in Finland since 1970, hospital outpatient care since 1998, and primary care since 2011. In the main analysis, self-harm episodes were identified using the following relevant codes in ICD-10 and corresponding codes in the Ninth Revision (ICD-9; used in 1987–1995) and the International Classification of Primary Care, Second edition (ICPC-2; used in some primary care facilities): intentional self-harm (X60–X84), event of undetermined intent (Y10–Y34), sequelae of intentional self-harm (Y87.0), and sequelae of events of undetermined intent (Y87.2). In a sensitivity analysis, we additionally included the following codes: other problems related to lifestyle, including self-damaging behaviour (Z72.8), and personal history of self-harm (Z91.5). A record associated with these codes indicates that an individual's injuries required somatic treatment or contributed to a psychiatric admission. We opted to include injuries and poisonings of undetermined intent (codes Y10–Y34), in line with previous investigations that utilised registry or electronic health record data,^25^^,^^26^ as it is widely acknowledged that a large proportion of these episodes will have entailed self-harm. Individuals who died by suicide before completing ninth grade did not apply for upper secondary education and are therefore not included in the Joint Application register. We did not examine death by suicide as a separate outcome due to lack of statistical power, and we opted not to examine non-fatal and fatal self-harm episodes as a coalesced single outcome as we consider them to be distinct phenomena that should not be examined in this manner.

### Covariates

We included the following demographic, socioeconomic, educational and intergenerational variables known to be associated with both self-harming behaviour and peer group composition as covariates: sex, birth year, number of schoolmates in the same grade, degree of urbanicity in residential location (urban, semi-urban, or rural) based on the urban-rural classification of the Finnish Environment Institute, morbidity index of the municipality by the Finnish Institute for Health and Welfare in quintiles (since data for 2001 were unavailable, we used data from 2002 instead), proportion of people without upper secondary or higher education in the postal code area in quintiles, proportion of unemployed people in the postal code area in quintiles, parental education level at the start of follow-up (primary, upper secondary, or higher education), parental income level in quintiles relative to the study population at the start of follow-up, and parental mental health and self-harm history at the start of follow-up (any prior mental disorder diagnosis [F00–F99] or recorded self-harm episode [X60–X84, Y10–Y34, Y87.0, Y87.2, Z72.8, Z91.5]).

Sex and birth year were measured at the individual level; urbanicity, morbidity, education, employment at the area level; number of same-grade schoolmates at the school level; and parental education, income, and self-harm and mental health history at the family level. Sex, birth year, urbanicity, education, income, employment, and parent-child links were retrieved from the full Finnish Population Register (FOLK) maintained by Statistics Finland, which contains yearly updated administrative records for all permanent residents. Postal codes were obtained from Statistics Finland. The number of schoolmates was calculated based on secondary school applications in the National Joint Application Register maintained by Statistics Finland. Mental health and self-harm diagnoses were acquired from the Care Register for Health Care and Register of Primary Health Care Visits of the Finnish Institute of Health and Welfare. The morbidity index is publicly available at https://sotkanet.fi/sotkanet/en/haku?

The median population sizes of Finnish municipalities and postal code areas in 2001–2016 were 6235 and 498 inhabitants, respectively. Missing values for area-level urbanicity and parental income were coded as “unknown” categories in the covariates and missing values in parental education denote primary education. Missing values in municipality (n = 543) and postal code areas (n = 8321) were derived based on the school's location (i.e., municipality or postal code area of other students attending the same school for whom the information was available) as the lower secondary school a student attends is typically the one closest to their residential location in Finland.

### Statistical analysis

We used mixed-effects Cox proportional hazards models to estimate the association between exposure to same-grade schoolmates with a self-harm episode recorded between first and ninth grade (i.e., from August 1 of the year they turned seven until completing ninth grade on May 31, around age 16) and the risk of subsequent self-harm episode among exposed cohort members. Cohort members without a recorded self-harm episode by the end of ninth grade were followed from June 1 of the year they completed ninth grade until first recorded self-harm episode, emigration, death, or December 31, 2020, whichever came first. We included a random intercept for each school to account for unobserved school-level variation in self-harm risk, including contextual differences in mental health prevalence. The associations were estimated as hazard ratios (HRs). In the main analyses, the Cox proportional hazards models were adjusted for sex, birth year, number of schoolmates in the same grade, area-level urbanicity, area-level morbidity, area-level education, area-level employment, parental education, parental income, and parental self-harm and mental health history. The model fit was evaluated using the Akaike Information Criterion (AIC).

As a secondary exposure variable in the main analysis, we categorised the number of schoolmates with a prior self-harm episode into three levels (none, one, or two or more). Additionally, to analyse the time-dependence of the associations, we estimated HRs for self-harm in different time windows following peer exposure by fitting separate Cox proportional hazards models for each 1-year period during the first 6 years of follow-up. The models were estimated independently for each time window rather than via a single model with time-varying covariates or interaction terms. This approach allowed us to examine how the strength of the association between peer exposure and self-harm varied across time since exposure. For each time window, we fitted two separate models: one adjusted for sex and birth year, and another adjusted for all the covariates.

To further scrutinise the time-dependence of the HRs, we repeated the time-windowed analysis using the end of seventh and eighth grade, in addition to ninth grade, as starting points for follow-up. To prevent overlap in exposure, we limited self-harm episodes used as exposure for the eighth and ninth grade peer networks to those recorded within the year preceding the end of each respective grade. In a sensitivity analysis, we estimated the Cox models using both a stricter and a broader classification of self-harm (i.e., a stricter classification using only codes X60–X84 and a broader classification including the additional codes Z72.8 and Z91.5). In another sensitivity analysis, we stratified the 2001–2016 study period into two shorter periods: 2001–2008 and 2009–2016. The statistical analyses were conducted using Stata (v16.1) and R Statistical Software (v4.2.2; R Core Team 2021) survival (v3.4.0)^27^ and coxme (v2.2.18.1)^28^ packages.

### Ethics approval

The Ethics Committee of the Finnish Institute for Health and Welfare approved the study plan (THL/184/6.02.01/2023§933). Data were linked with the permission of Statistics Finland (TK-53-1696-16) and the Finnish Institute of Health and Welfare. According to Finnish law, informed consent is not required for register-based studies.

### Role of the funding source

The funders had no role in study design, data collection, data Formal analysis, data interpretation, or writing of the report.

## Results

Table 1 shows descriptive statistics of the study population (for additional descriptive statistics, see Supplementary Table S1, and for sex-disaggregated descriptive statistics, see Supplementary Table S2). Among the 11,378 ninth-grade peer networks, 1710 (15.0%) had at least one cohort member with a recorded self-harm episode (ICD-10 codes X60–X84, Y10–Y34, Y87.0, Y87.2) at the start of follow-up (1466 [12.9%] had one and 244 [2.1%] had two or more). Median age at the start of follow-up was 16.0 (IQR, 15.7–16.2) years. For those exposed to peer's self-harm, the median length was 9.6 (IQR, 6.6–13.6) years, and for the unexposed, the median length was 12.6 (IQR, 8.6–16.6) years. The difference in the median between the exposure groups is largely explained by the larger number of recorded self-harm episodes in the peer networks in the later birth cohorts. That is, more individuals in these cohorts were exposed, and because follow-up ended at the latest in 2020, they were followed for a shorter period.

**Table 1:** Descriptive statistics of the study population. Information is presented separately for cohort members who self-harmed during grades 1–9 and those who had not self-harmed by the end of grade 9.

Characteristic	Self-harm episode during grades 1–9		No self-harm episode by the end of grade 9	
	n	%	n	%
Sex				
Male	799	35.15	464,085	51.03
Female	1474	64.85	445,438	48.97
Birth year				
1985	84	3.70	57,963	6.37
1986	89	3.92	56,540	6.22
1987	83	3.65	55,722	6.13
1988	87	3.83	58,588	6.44
1989	101	4.44	58,598	6.44
1990	93	4.09	60,435	6.64
1991	106	4.66	59,715	6.57
1992	103	4.53	59,966	6.59
1993	134	5.90	59,436	6.53
1994	163	7.17	59,954	6.59
1995	156	6.86	58,551	6.44
1996	195	8.58	56,104	6.17
1997	220	9.68	54,514	5.99
1998	190	8.36	51,939	5.71
1999	237	10.43	52,544	5.78
2000	232	10.21	48,954	5.38
Recorded self-harm episode				
X60–X84 Intentional self-harm	1465	64.45	13,914	1.53
Y10–Y34 Event of undetermined intent	653	28.73	4583	0.50
Y87.0 Sequelae of intentional self-harm	6	0.26	62	0.01
Y87.2 Sequelae of events of undetermined intent	10	0.44	42	0.00
aZ72.8 Other problems related to lifestyle, including self-damaging behaviour	251	11.04	1377	0.15
aZ91.5 Personal history of self-harm	115	5.06	634	0.07
Any of the above	2273	100.00	19,009	2.09

a Used in sensitivity analysis only.

During 10.9 million person-years of follow-up, with a median length of 11.6 (IQR, 7.8–15.6) years, 17,897 cohort members self-harmed, with an incidence rate of 165 per 100,000 person-years at risk. Of the 17,897 cohort members with a recorded self-harm episode during follow-up, 8667 were male and 9230 were female, with incidence rates of 156.3 and 173.7 per 100,000 person-years at risk, respectively. For the number of self-harm cases and incidence rates during follow-up stratified by all the covariates, see Supplementary Table S3. For the number of self-harm episodes by school grade, see Supplementary Table S4.

**Fig. 2:**
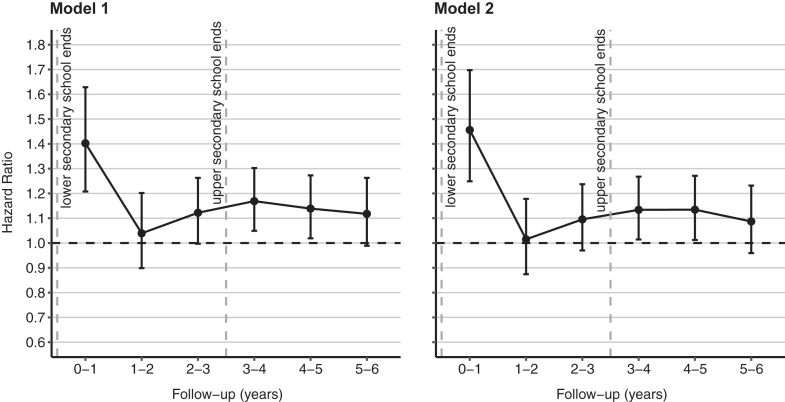
**Associations between having ninth-grade schoolmates who had self-harmed and subsequent self-harm risk.** The associations are shown in 1-year time windows for the first 6 years of follow-up. The error bars represent the 95% confidence interval. The Cox proportional hazards models were estimated independently for each time window. In Model 1, the models were adjusted for sex and birth year. In Model 2, the models were adjusted for sex, birth year, number of schoolmates in the same grade, area-level urbanicity, area-level morbidity, area-level education, area-level employment, parental education, parental income, and parental self-harm and mental health history, with a random intercept per school.

Table 2 shows results for the association between having schoolmates who had self-harmed and subsequent self-harm risk. In a model adjusted only for sex and birth year, exposure to ninth-grade schoolmates who had self-harmed was associated with a 10% higher rate of subsequent self-harm during follow-up compared to unexposed cohort members (HR 1.10, 95% CI 1.06–1.14). When adjusting for all the covariates, the rate of subsequent self-harm was 5% higher for the exposed (HR 1.05, 95% CI 1.01–1.09). The random intercepts for schools (i.e., school-specific excess risk of self-harm) in the mixed-effects Cox proportional hazards model adjusted for all the covariates had a standard deviation of 0.25, indicating that students in a school one standard deviation above the mean had a 28% higher rate of subsequently self-harming (e^0.25^ = 1.28). Schoenfeld residual-based test indicated that the proportional hazards assumption was not valid when considering the entire follow-up (χ^2^ = 6.0, P = 0.01). Fig. 2 shows the HRs for the associations in 1-year time windows for the first 6 years of follow-up. When adjusting only for sex and birth year, the rate of self-harm for the exposed was highest during the first year of follow-up (HR 1.40, 95% CI 1.21–1.63) and higher also during the fourth (HR 1.17, 95% CI 1.05–1.30) and fifth year of follow-up (HR 1.14, 95% CI 1.02–1.27) compared to the unexposed. When adjusting for all the covariates, the rates were similarly higher in the first (HR 1.46, 95% CI 1.25–1.70), fourth (HR 1.13, 95% CI 1.01–1.27), and fifth year of follow-up (HR 1.13, 95% CI 1.01–1.27). The AIC evaluation indicated a better fit for the model adjusted for all the covariates.

**Table 2:** Associations between having schoolmates who had self-harmed and subsequent self-harm risk.

Follow-up period	N	Number of schoolmates who had self-harmed (exposure)						
		None (ref.)	One or more		One		Two or more	
		n	n	HR (95% CI)	n	HR (95% CI)	n	HR (95% CI)
Entire follow-up	909,523	14,560	3337	1.05 (1.01–1.09)	2763	1.03 (0.99–1.07)	574	1.18 (1.08–1.28)
Year 1	909,523	683	243	1.46 (1.25–1.70)	194	1.40 (1.19–1.65)	49	1.75 (1.30–2.37)
Year 2	908,137	900	240	1.01 (0.87–1.18)	188	0.97 (0.83–1.14)	52	1.23 (0.92–1.65)
Year 3	906,530	1292	367	1.10 (0.97–1.24)	300	1.09 (0.96–1.24)	67	1.11 (0.86–1.44)
Year 4	904,240	1525	441	1.13 (1.01–1.27)	351	1.09 (0.97–1.23)	90	1.34 (1.07–1.68)
Year 5	849,379	1547	413	1.13 (1.01–1.27)	332	1.08 (0.96–1.23)	81	1.43 (1.13–1.80)
Year 6	793,948	1378	330	1.09 (0.96–1.23)	264	1.02 (0.89–1.17)	66	1.48 (1.15–1.91)

Hazard ratios (HRs) with 95% confidence intervals shown for the entire follow-up and separately in 1-year time windows for the first 6 years of follow-up. Results are shown for the main analysis using a dichotomised exposure (none vs. one or more) and for the analysis using a three-level dose-response exposure (none vs. one and none vs. two or more). The capital N denotes the total number cohort members in the analysis and the lowercase n's denote the number of cohort members who self-harmed during the follow-up period, stratified by the exposure. The Cox proportional hazards models were estimated independently for each time window and adjusted for sex, birth year, number of schoolmates in the same grade, area-level urbanicity, area-level morbidity, area-level education, area-level employment, parental education, parental income, and parental self-harm and mental health history, with a random intercept per school.

The results remained similar when using the stricter classification of self-harming behaviour (i.e., using only codes X60–X84) and when using the broader classification (i.e., including the additional Z72.8 and Z91.5 codes), both showing highest self-harm risk during the first year of follow-up for the exposed (Supplementary Figure S1 and Supplementary Table S5). We also examined differences in the 2001–2016 study period by stratifying it into two shorter periods (2001–2008 and 2009–2016; Supplementary Figure S2 and Supplementary Table S6). When considering the entire follow-up, the risk was higher only for the exposed who were in the ninth grade during 2009–2016 (HR 1.09, 95% CI 1.03–1.15). However, during the first year of follow-up, the risk was similarly higher in both periods (2001–2008: HR 1.46, 95% CI 1.11–1.92; 2009–2016: HR 1.48, 95% CI 1.23–1.78).

Using more granular dose-response exposure categorisation, there was no evidence that having one ninth-grade schoolmate who had self-harmed was associated with greater risk when considering the entire follow-up (HR 1.03, 95% CI 0.99–1.07), yet having two or more schoolmates who had self-harmed was associated with 18% higher rate of a subsequent self-harm (HR 1.18, 95% CI 1.08–1.28). During the first year of follow-up, having one ninth-grade schoolmate who had self-harmed was associated with 40% higher rate (HR 1.40, 95% CI 1.19–1.65) and having two or more was associated with 75% higher rate of a subsequent self-harm (HR 1.75, 95% CI 1.30–2.37).

We repeated the time-windowed analysis using the end of seventh, eighth, and ninth grade as starting points for follow-up (with non-overlapping exposure periods). The descriptive statistics of the study population at the end of seventh and eighth grade are shown in Supplementary Table S7, and the results are shown in Supplementary Figure S3 and Supplementary Table S8. The rate of self-harm was 49% higher in the first year of follow-up for those with ninth-grade schoolmates who had self-harmed during the ninth grade (HR 1.49, 95% CI 1.21–1.82). For those with eighth-grade schoolmates who had self-harmed during the eighth grade, the rate was highest during the second year (HR 1.36, 95% CI 1.07–1.74) and higher also during the fifth year of follow-up (HR 1.29, 95% CI 1.09–1.53). For students with seventh-grade schoolmates who had self-harmed by the end of seventh grade, the self-harm rate was not significantly higher (at P < 0.05) in any time window. However, in the third year of follow-up, the rate was close to statistical significance (HR 1.25, 95% CI 1.00–1.57, P = 0.051).

## Discussion

In this analysis of Finnish nationwide registry data covering over 900,000 individuals in 909 lower secondary schools, having schoolmates who had self-harmed was associated with a higher rate of subsequent self-harm. The rate was not consistent over time but was highest during the early follow-up period. The observed associations were not explained by differences in area-level factors, such as urbanicity, general morbidity, or socioeconomic characteristics, nor by parental history of self-harm or mental disorders, or by parental socioeconomic position.

To our knowledge, the present study is the largest investigation on this topic to date. Our findings are consistent with previous survey studies reporting associations between exposure to adolescent peers' self-harming behaviour and subsequent higher self-harm risk.^16^, ^17^, ^18^, ^19^, ^20^, ^21^ The observed association between having ninth-grade schoolmates who had self-harmed and subsequent higher self-harm risk among exposed individuals was greatest in the first year of follow-up when the cohort members were around age 16. The exploratory analysis, using the end of seventh, eighth, and ninth grades as starting points for follow-up (with non-overlapping exposure), showed that the risk of subsequent self-harm for the exposed was most prominent in the third, second, and first years of follow-up, respectively, all coinciding with age around 16. Since age 16 typically marks the beginning of upper secondary education in Finland—a period characterised by increased academic pressure, shifting peer relationships, and intensified challenges related to identity formation—our findings suggest that peer exposure may exacerbate these existing stressors. The observed drop in risk at age around 17 may reflect a period of relative stability following the significant transitions at age 16. By this point, students are more familiar with academic expectations and social dynamics, which may reduce stress and peer-related vulnerability. Similar results where stressful life events modify the association between exposure to a peer's self-harming behaviour and subsequent self-harm risk have been reported previously.^18^^,^^20^ Differences in educational pathways (e.g., academic vs. vocational) may also partly mediate the observed associations.

Several mechanisms could potentially explain these observed associations, including non-causal explanations such as residual confounding, and causal effects such as direct social transmission of self-harming behaviour within peer networks through personal acquaintance with a self-harming individual.^29^ Peer networks can exert peer influence also through broader mechanisms such as normative expectations or observational learning, even without close personal ties. It should also be acknowledged that what may be transmitted through peer exposure is not necessarily the behaviour of self-harm itself, but rather shared emotional distress, coping behaviours, or the normalisation of self-harm, all of which could contribute to the development of self-harming behaviour. For instance, exposure to peers’ self-harming behaviours may normalise them, leading to the perception that self-harm is an acceptable coping strategy.^15^ Because registry-recorded self-harm episodes following presentation to healthcare services were used as the measure of self-harming behaviour in this study, the observed associations might also be explained by normalisation where individuals who have self-harmed exhibit a lowered threshold for seeking healthcare when peers within the same social network have self-harmed and subsequently presented to healthcare services, particularly in cases of less severe self-harm.

The main strengths of the present study are examination of a nationwide study population, inclusion of interlinked registry data from both primary and secondary health care, and institutionally imposed peer networks in a lower secondary school setting. A major strength of utilising population-wide interlinked registers to conduct epidemiological investigations is that they are unaffected by health-related selection and non-participation biases, which often compromise the validity of survey-based cohort studies. Additionally, defining institutionally imposed social networks, such as schoolmates, employing the registry data mitigates self-selection bias arising from assortative relating or homophily.^22^ Nevertheless, it is important to interpret our findings in relation to the study's limitations.

First, although we accounted for numerous covariates, residual confounding due to unmeasured or inaccurately measured variables cannot be entirely ruled out. Relatedly, even though educational tracks (e.g., academic vs. vocational) following lower secondary school completion are likely associated with differences in social environment and health—factors that may relate to self-harm risk—we chose not to control for these variables. This is because school application outcomes and track choices are not fully predetermined, as they are influenced by earlier academic performance, social environment, and health status—all of which may already reflect the early development of self-harming behaviours or their precursors. Including school type or educational attainment as covariates could therefore introduce post-treatment bias by conditioning on consequences of prior exposures. That said, it should be acknowledged that differences in educational pathways may also partly mediate the observed associations.

Second, as registry-recorded episodes following presentation to healthcare services was the measure of self-harm, it should be recognised that self-harming individuals who do present to services represent only a small minority of all individuals who engage in self-harm and may not accurately represent the broader population of people who self-harm.^13^^,^^30^ Third, although the Finnish healthcare registers are considered to have a good diagnostic validity,^31^ there is likely both regional and temporal variation in coverage. For example, while data on primary healthcare was available from 2011, its coverage was initially incomplete. With temporal improvement in coverage, a larger proportion of diagnoses is recorded in the registers at the end of follow-up compared to the beginning. Fourth, we recognise that the dynamics between self-harming behaviour and mental health symptoms within peer networks may have influenced the associations observed in this study, as either can precede or contribute to the other. For example, exposure to a peer's self-harming behaviour may negatively affect an individual's mental health, while an individual's mental health symptoms may, in turn, influence the self-harming behaviour of peers. We encourage future research with more appropriate research paradigms to explore these dynamics within adolescent peer networks.

Fifth, while our approach assumes a high probability of social interaction among same-grade schoolmates, it cannot account for the presence or quality of specific ties, such as friendships or cliques, in which behaviour transmission is more likely. As such, our findings reflect broader social exposure to self-harming behaviours among same-grade schoolmates rather than confirmed peer influence, and conclusions about possible social transmission of self-harming behaviour through peer networks should be interpreted in the context of these limitations. Also, modelling peer effects only at the level of school year or grade may oversimplify the social structure by ignoring variation within classes. However, given that students maintain significant connections both within their classes and across the entire grade, we believe this approach provides a meaningful and robust representation of peer influences. Moreover, as self-harm episodes are often relatively overt and noticeable events, their impact is more likely to extend across the entire school year cohort, further supporting the validity of our broader school-grade approach. Relatedly, a small proportion of same-grade schoolmates were siblings, which could have had a minor influence on the observed estimates. However, due to their marginal proportion in the cohort, this is unlikely to alter the interpretation of the findings. Finally, since Finland is a small Nordic country with a relatively homogeneous population, replications in other countries are needed to establish the generalisability of our findings.

Our results, based on a nationwide cohort of over 900,000 Finnish people, showed that having schoolmates in secondary school who had self-harmed is associated with a higher risk of subsequent self-harm, suggesting that self-harming behaviour may be socially transmitted within adolescent peer networks. These findings highlight the important role of primary care and schools in the early intervention of self-harming behaviour and in preventing its possible transmission among adolescents. Proactive plans should be developed for schools to effectively identify and address self-harming behaviour.^32^ These plans should prepare school staff to identify self-harm and address underlying risk factors, such as harmful social media usage and bullying.^33^^,^^34^ Innovative web-based training may equip staff with the requisite knowledge and skills to provide effective support following a self-harm episode to the affected student and to their schoolmates, and also to promote whole-school self-harm prevention approaches.^35^ Further research is needed to develop a comprehensive and robust evidence-base for the provision of structured prevention programmes in secondary schools.^36^ Additionally, efforts should be made to improve access to preventive services and therapies in primary care and school settings, emphasising peer support and lived experience in the delivery of services, while enhancing collaboration between healthcare, education, social services, and media to create a comprehensive support system for at-risk adolescents.^37^^,^^38^

## Contributors

JA, RTW, NK, MG, RN, and CH conceived the research question. JA, MG, and RN conducted the statistical analyses. JA and CH wrote first draft of the manuscript. CH and ME obtained funding. CH provided supervision. JA and CH accessed and verified all the data in the study. All authors critically reviewed and edited the manuscript. All authors had access to presented and output data and had final responsibility for the decision to submit for publication.

## Data sharing statement

Data for the present study is property of Statistics Finland and Finnish Institute of Health and Welfare. The data are available from these authorities (for more information, see www.findata.fi and www.stat.fi), but restrictions apply.

## Declaration of interests

NK is a committee member for guideline groups for the National Institute for Health and Care Excellence (NICE); an advisory group member for the Department of Health and Social Care (DHSC); and reports grants from DHSC, the National Institute for Health and Care Research (NIHR), and the Healthcare Quality Improvement Partnership. All other authors declare no competing interests.

## References

[1] Knipe D., Padmanathan P., Newton-Howes G., Chan L.F., Kapur N. (2022). Suicide and self-harm. Lancet.

[2] Skegg K. (2005). Self-harm. Lancet.

[3] Muehlenkamp J.J., Claes L., Havertape L., Plener P.L. (2012). International prevalence of adolescent non-suicidal self-injury and deliberate self-harm. Child Adolesc Psychiatry Ment Health.

[4] Swannell S.V., Martin G.E., Page A., Hasking P., St John N.J. (2014). Prevalence of nonsuicidal self-injury in nonclinical samples: systematic review, meta-analysis and meta-regression. Suicide Life Threat Behav.

[5] Xiao Q.Q., Song X.Z., Huang L.J., Hou D.D., Huang X.H. (2022). Global prevalence and characteristics of non-suicidal self-injury between 2010 and 2021 among a non-clinical sample of adolescents: a meta-analysis. Front Psychiatry.

[6] Cooper J., Kapur N., Webb R. (2005). Suicide after deliberate self-harm: a 4-year cohort study. Am J Psychiatry.

[7] Morgan C., Webb R.T., Carr M.J. (2017). Incidence, clinical management, and mortality risk following self harm among children and adolescents: cohort study in primary care. BMJ.

[8] Mars B., Heron J., Klonsky E.D. (2019). Predictors of future suicide attempt among adolescents with suicidal thoughts or non-suicidal self-harm: a population-based birth cohort study. Lancet Psychiatry.

[9] Kiviruusu O., Ranta K., Lindgren M. (2024). Mental health after the COVID-19 pandemic among Finnish youth: a repeated, cross-sectional, population-based study. Lancet Psychiatry.

[10] Trafford A.M., Carr M.J., Ashcroft D.M. (2023). Temporal trends in eating disorder and self-harm incidence rates among adolescents and young adults in the UK in the 2 years since onset of the COVID-19 pandemic: a population-based study. Lancet Child Adolesc Health.

[11] Steeg S., John A., Gunnell D.J. (2022). The impact of the COVID-19 pandemic on presentations to health services following self-harm: systematic review. Br J Psychiatry.

[12] Bommersbach T.J., Olfson M., Rhee T.G. (2024). National trends in emergency department visits for suicide attempts and intentional self-harm. Am J Psychiatry.

[13] Hawton K., Saunders K.E., O'Connor R.C. (2012). Self-harm and suicide in adolescents. Lancet.

[14] McEvoy D., Brannigan R., Cooke L. (2023). Risk and protective factors for self-harm in adolescents and young adults: an umbrella review of systematic reviews. J Psychiatr Res.

[15] Hawton K., Hill N.T.M., Gould M., John A., Lascelles K., Robinson J. (2020). Clustering of suicides in children and adolescents. Lancet Child Adolesc Health.

[16] Abrutyn S., Mueller A.S. (2014). Are suicidal behaviors contagious in adolescence? Using longitudinal data to examine suicide suggestion. Am Sociol Rev.

[17] Randall J.R., Nickel N.C., Colman I. (2015). Contagion from peer suicidal behavior in a representative sample of American adolescents. J Affect Disorders.

[18] Swanson S.A., Colman I. (2013). Association between exposure to suicide and suicidality outcomes in youth. Can Med Assoc J.

[19] Feigelman W., Gorman B.S. (2008). Assessing the effects of peer suicide on youth suicide. Suicide Life Threat Behav.

[20] Gould M.S., Lake A.M., Kleinman M., Galfalvy H., Chowdhury S., Madnick A. (2018). Exposure to suicide in high schools: impact on serious suicidal ideation/behavior, depression, maladaptive coping strategies, and attitudes toward help-seeking. Int J Environ Res Public Health.

[21] Crudgington H., Wilson E., Copeland M., Morgan C., Knowles G. (2023). Peer-friendship networks and self-injurious thoughts and behaviors in adolescence: a systematic review of sociometric school-based studies that use social network analysis. Adolesc Res Rev.

[22] McPherson M., Smith-Lovin L., Cook J.M. (2001). Birds of a feather: homophily in social networks. Annu Rev Sociol.

[23] Alho J., Gutvilig M., Niemi R. (2024). Transmission of mental disorders in adolescent peer networks. JAMA Psychiatry.

[24] Brown B.B., Larson J. (2009). Peer relationships in adolescence. Handb Adolesc Psychol.

[25] Mars B., Cornish R., Heron J. (2016). Using data linkage to investigate inconsistent reporting of self-harm and questionnaire non-response. Arch Suicide Res.

[26] Tidemalm D., Beckman K., Dahlin M. (2015). Age-specific suicide mortality following non-fatal self-harm: national cohort study in Sweden. Psychol Med.

[27] Therneau T. (2022). A Package for Survival Analysis in R. [R package version 3.4-0]. https://CRAN.R-project.org/package=survival.

[28] Therneau T.M. (2022). Mixed Effects Cox Models [R package coxme version 2.2-18.1]. https://CRAN.R-project.org/package=coxme.

[29] Gould M.S., Davidson L. (1988). Suicide contagion among adolescents. Adv Adolesc Ment Health.

[30] Geulayov G., Casey D., McDonald K.C. (2018). Incidence of suicide, hospital-presenting non-fatal self-harm, and community-occurring non-fatal self-harm in adolescents in England (the iceberg model of self-harm): a retrospective study. Lancet Psychiatry.

[31] Sund R. (2012). Quality of the Finnish hospital discharge register: a systematic review. Scand J Publ Health.

[32] Hawton K., Lascelles K., Stewart A. (2015). Identifying and responding to suicide clusters and contagion: a practical resource. London: Public Health England. https://assets.publishing.service.gov.uk/government/uploads/system/uploads/attachment_data/file/839621/PHE_Suicide_Cluster_Guide.pdf.

[33] Fisher H.L., Moffitt T.E., Houts R.M., Belsky D.W., Arseneault L., Caspi A. (2012). Bullying victimisation and risk of self harm in early adolescence: longitudinal cohort study. BMJ.

[34] John A., Glendenning A.C., Marchant A. (2018). Self-harm, suicidal behaviours, and cyberbullying in children and young people: systematic review. J Med Internet Res.

[35] Burn A.M., Hall P., Anderson J. (2024). A web-based training program for school staff to respond to self-harm: design and development of the supportive response to self-harm program. JMIR Form Res.

[36] Liljedahl S.I., Hellner C., Pettersson A., Ghaderi A. (2023). School-based self-harm prevention programs: a systematic review with implications for international implementation. Scand J Psychol.

[37] Vorma H. (2020). National Mental Health Strategy and Programme for Suicide Prevention 2020–2030. Helsinki: Ministry of Social Affairs and Health. https://julkaisut.valtioneuvosto.fi/bitstream/handle/10024/162234/STM_2020_15.pdf.

[38] Moran P., Chandler A., Dudgeon P. (2024). The Lancet Commission on self-harm. Lancet.

