# Supplementary material for: Examination of self-harm clustering in adolescent peer networks: a nationwide registry cohort study in Finland
Source: Lancet Reg Health Eur. 2025 Nov 2;60:101517. doi: 10.1016/j.lanepe.2025.101517 (PMC12621558; doi:10.1016/j.lanepe.2025.101517)
Supplement: Supplementary Material 1 [file mmc1.docx]

**Supplementary Material**

**CONTENTS**

[Methods S1: Structure of the Finnish comprehensive and upper secondary educational systems 2](#_Toc203735013)

[Table S1: Additional descriptive statistics of the study population. 3](#_Toc203735014)

[Table S2: All descriptive statistics disaggregated by sex. 5](#_Toc203735015)

[Table S3: Number of cases and incidence rates of cohort members with a recorded self-harm episode during follow-up stratified by the covariates. 7](#_Toc203735016)

[Table S4: Number of self-harm episodes by school grade. 9](#_Toc203735017)

[Table S5: Associations between having ninth-grade schoolmates who had self-harmed and subsequent self-harm risk, using both stricter and broader classification of self-harm. 10](#_Toc203735018)

[Table S6: Associations between having ninth-grade schoolmates who had self-harmed and subsequent self-harm risk, stratified into two time periods: 2001–2008 and 2009–2016. 11](#_Toc203735019)

[Table S7: Descriptive statistics of the study population at the end of eighth (A) and seventh (B) grade. 12](#_Toc203735020)

[Table S8: Associations between having same-grade schoolmates who self-harmed and the risk of subsequent self-harm in lower secondary school, with follow-up starting at the end of each respective grade. 16](#_Toc203735021)

[Figure S1: Associations between having ninth-grade schoolmates who had self-harmed and subsequent self-harm risk, using both stricter and broader classification of self-harm. 17](#_Toc203735022)

[Figure S2: Associations between having ninth-grade schoolmates who had self-harmed and subsequent self-harm risk, stratified into two time periods: 2001–2008 and 2009–2016. 18](#_Toc203735023)

[Figure S3: Associations between having same-grade schoolmates who self-harmed and the risk of subsequent self-harm in lower secondary school, with follow-up starting at the end of each grade. 19](#_Toc203735024)

## Methods S1: Structure of the Finnish comprehensive and upper secondary educational systems

In Finland, nine years of comprehensive education begins the year when children reach age seven and continues until approximately age 16. The comprehensive school system, which includes primary (grades 1–6) and lower secondary (grades 7–9) levels, is uniform, with no academic or vocational tracks. Most students attend the municipal school nearest to their home, and fewer than 2% opt for a private or publicly funded school outside their municipality.^1^

After completing comprehensive education, students may enter upper secondary education, which requires a comprehensive school certificate. Applications to upper secondary schools are processed through a joint system, with admission based on school grades. The upper secondary level includes academic and vocational tracks. General (academic) upper secondary education is organised around courses rather than traditional year classes and concludes with a nationally comparable matriculation examination. Vocational education, structured by year classes, prepares students for specific careers. Both tracks typically take three years to complete.

## Table S1: Additional descriptive statistics of the study population.

|  |  |  |  |  |
| --- | --- | --- | --- | --- |
|  | **Self-harm episode during grades 1–9** | | **No self-harm episode before end of grade 9** | |
| **Characteristic** | **n or mean** | **% or SD** | **n or mean** | **% or SD** |
| **Same-grade schoolmates with a recorded self-harm episode** |  |  |  |  |
| No | 204 | 8·97 | 738 184 | 81·16 |
| Yes | 2 069 | 91·03 | 171 339 | 18·84 |
| **Number of schoolmates in the same grade** | 110.03 | 48·30 | 112·50 | 47·85 |
| **Area-level urbanicity** |  |  |  |  |
| Unknown | 156 | 6·86 | 8 965 | 0·99 |
| Rural | 437 | 19·23 | 245 662 | 27·01 |
| Semi-urban | 375 | 16·50 | 170 694 | 18·77 |
| Urban | 1 305 | 57·41 | 484 202 | 53·24 |
| **Area-level morbidity** |  |  |  |  |
| Quintile 1 (lowest) | 550 | 24·20 | 182 448 | 20·06 |
| Quintile 2 | 507 | 22·31 | 182 868 | 20·11 |
| Quintile 3 | 420 | 18·48 | 181 746 | 19·98 |
| Quintile 4 | 459 | 20·19 | 180 649 | 19·86 |
| Quintile 5 (highest) | 337 | 14·83 | 181 812 | 19·99 |
| **Area-level employment** |  |  |  |  |
| Quintile 1 (highest) | 373 | 16·41 | 182 141 | 20·03 |
| Quintile 2 | 411 | 18·08 | 181 834 | 19·99 |
| Quintile 3 | 472 | 20·77 | 181 971 | 20·01 |
| Quintile 4 | 499 | 21·95 | 182 008 | 20·01 |
| Quintile 5 (lowest) | 518 | 22·79 | 181 569 | 19·96 |
| **Area-level education** |  |  |  |  |
| Quintile 1 (highest) | 643 | 28·29 | 181 716 | 19·98 |
| Quintile 2 | 493 | 21·69 | 181 848 | 19·99 |
| Quintile 3 | 473 | 20·81 | 181 973 | 20·01 |
| Quintile 4 | 362 | 15·93 | 181 939 | 20·00 |
| Quintile 5 (lowest) | 302 | 13·29 | 182 047 | 20·02 |
| **Mother’s education** |  |  |  |  |
| Primary | 447 | 19·67 | 122 707 | 13·49 |
| Secondary | 1 048 | 46·11 | 388 493 | 42·71 |
| Higher | 778 | 34·23 | 398 323 | 43·79 |
| **Father’s education** |  |  |  |  |
| Primary | 700 | 30·80 | 208 856 | 22·96 |
| Secondary | 993 | 43·69 | 397 897 | 43·75 |
| Higher | 580 | 25·52 | 302 770 | 33·29 |
| **Mother’s income** |  |  |  |  |
| Unknown | 67 | 2·95 | 12 207 | 1·34 |
| Quintile 1 (lowest) | 598 | 26·31 | 179 287 | 19·71 |
| Quintile 2 | 406 | 17·86 | 179 501 | 19·74 |
| Quintile 3 | 381 | 16·76 | 179 527 | 19·74 |
| Quintile 4 | 426 | 18·74 | 179 485 | 19·73 |
| Quintile 5 (highest) | 395 | 17·38 | 179 516 | 19·74 |
| **Father’s income** |  |  |  |  |
| Unknown | 241 | 10·60 | 45 667 | 5·02 |
| Quintile 1 (lowest) | 569 | 25·03 | 172 589 | 18·98 |
| Quintile 2 | 348 | 15·31 | 172 839 | 19·00 |
| Quintile 3 | 375 | 16·50 | 172 808 | 19·00 |
| Quintile 4 | 380 | 16·72 | 172 801 | 19·00 |
| Quintile 5 (highest) | 360 | 15·84 | 172 819 | 19·00 |
| **Mother’s self-harm or mental disorder** |  |  |  |  |
| No | 1 568 | 68·98 | 794 266 | 87·33 |
| Yes | 705 | 31·02 | 115 257 | 12·67 |
| **Father’s self-harm or mental disorder** |  |  |  |  |
| No | 1 705 | 75·01 | 798 726 | 87·82 |
| Yes | 568 | 24·99 | 110 797 | 12·18 |

## Table S2: All descriptive statistics disaggregated by sex.

|  | **Males** |  | **Females** |  |
| --- | --- | --- | --- | --- |
| **Characteristic** | n | **%** | n | **%** |
| **Birth year** |  |  |  |  |
| 1985 | 29 571 | 6·36 | 28 482 | 6·37 |
| 1986 | 28 934 | 6·22 | 27 701 | 6·20 |
| 1987 | 28 562 | 6·14 | 27 249 | 6·10 |
| 1988 | 29 894 | 6·43 | 28 792 | 6·44 |
| 1989 | 30 022 | 6·46 | 28 683 | 6·42 |
| 1990 | 30 962 | 6·66 | 29 570 | 6·62 |
| 1991 | 30 396 | 6·54 | 29 436 | 6·59 |
| 1992 | 30 674 | 6·60 | 29 401 | 6·58 |
| 1993 | 30 313 | 6·52 | 29 274 | 6·55 |
| 1994 | 30 601 | 6·58 | 29 531 | 6·61 |
| 1995 | 29 977 | 6·45 | 28 749 | 6·43 |
| 1996 | 28 866 | 6·21 | 27 451 | 6·14 |
| 1997 | 27 827 | 5·98 | 26 930 | 6·02 |
| 1998 | 26 539 | 5·71 | 25 606 | 5·73 |
| 1999 | 26 971 | 5·80 | 25 835 | 5·78 |
| 2000 | 24 899 | 5·35 | 24 313 | 5·44 |
| **Recorded self-harm episode** |  |  |  |  |
| X60-X84 Intentional self-harm | 6 361 | 1·37 | 9 063 | 2·03 |
| Y10-Y34 Event of undetermined intent | 3 557 | 0·76 | 1 855 | 0·41 |
| Y87.0 Sequelae of intentional self-harm | 26 | 0·01 | 43 | 0·01 |
| Y87.2 Sequelae of events of undetermined intent | 32 | 0·01 | 21 | 0·00 |
| *Z72.8 Other problems related to lifestyle, including self-damaging behaviour | 679 | 0·15 | 952 | 0·21 |
| *Z91.5 Personal history of self-harm | 292 | 0·06 | 458 | 0·10 |
| Any of the above | 10 122 | 2·18 | 11 375 | 2·54 |
| **Same-grade schoolmates with a recorded self-harm episode** |  |  |  |  |
| No | 376 481 | 80·96 | 362 073 | 81·00 |
| Yes | 88 527 | 19·04 | 84 930 | 19·00 |
| **Number of same-grade schoolmates** | 112.14 | 47·99 | 112·85 | 47·71 |
| **Area-level urbanicity** |  |  |  |  |
| Unknown | 4 627 | 1·00 | 4 506 | 1·01 |
| Rural | 129 532 | 27·86 | 116 621 | 26·09 |
| Semi-urban | 88 181 | 18·96 | 82 924 | 18·55 |
| Urban | 242 668 | 52·19 | 242 952 | 54·35 |
| **Area-level morbidity** |  |  |  |  |
| Quintile 1 (lowest) | 93 511 | 20·11 | 89 548 | 20·03 |
| Quintile 2 | 93 852 | 20·18 | 89 572 | 20·04 |
| Quintile 3 | 92 671 | 19·93 | 89 534 | 20·03 |
| Quintile 4 | 92 172 | 19·82 | 88 971 | 19·90 |
| Quintile 5 (highest) | 92 802 | 19·96 | 89 378 | 19·99 |
| **Area-level employment** |  |  |  |  |
| Quintile 1 (highest) | 93 706 | 20·15 | 88 848 | 19·88 |
| Quintile 2 | 92 900 | 19·98 | 89 387 | 20·00 |
| Quintile 3 | 92 993 | 20·00 | 89 497 | 20·02 |
| Quintile 4 | 93 161 | 20·03 | 89 392 | 20·00 |
| Quintile 5 (lowest) | 92 248 | 19·84 | 89 879 | 20·11 |
| **Area-level education** |  |  |  |  |
| Quintile 1 (highest) | 91 260 | 19·63 | 91 160 | 20·39 |
| Quintile 2 | 93 190 | 20·04 | 89 204 | 19·96 |
| Quintile 3 | 93 329 | 20·07 | 89 163 | 19·95 |
| Quintile 4 | 93 433 | 20·09 | 88 905 | 19·89 |
| Quintile 5 (lowest) | 93 796 | 20·17 | 88 571 | 19·81 |
| **Mother's education** |  |  |  |  |
| Primary | 62 902 | 13·53 | 60 296 | 13·49 |
| Secondary | 198 479 | 42·68 | 191 156 | 42·76 |
| Higher | 203 627 | 43·79 | 195 551 | 43·75 |
| **Father's education** |  |  |  |  |
| Primary | 106 164 | 22·83 | 103 458 | 23·14 |
| Secondary | 203 863 | 43·84 | 195 121 | 43·65 |
| Higher | 154 981 | 33·33 | 148 424 | 33·20 |
| **Mother's income** |  |  |  |  |
| Unknown | 6 268 | 1·35 | 6 008 | 1·34 |
| Quintile 1 (lowest) | 91 989 | 19·78 | 87 960 | 19·68 |
| Quintile 2 | 91 652 | 19·71 | 88 293 | 19·75 |
| Quintile 3 | 91 591 | 19·70 | 88 356 | 19·77 |
| Quintile 4 | 91 640 | 19·71 | 88 307 | 19·76 |
| Quintile 5 (highest) | 91 868 | 19·76 | 88 079 | 19·70 |
| **Father's income** |  |  |  |  |
| Unknown | 23 163 | 4·98 | 22 759 | 5·09 |
| Quintile 1 (lowest) | 88 021 | 18·93 | 85 197 | 19·06 |
| Quintile 2 | 88 734 | 19·08 | 84 484 | 18·90 |
| Quintile 3 | 88 745 | 19·08 | 84 473 | 18·90 |
| Quintile 4 | 88 200 | 18·97 | 85 019 | 19·02 |
| Quintile 5 (highest) | 88 145 | 18·96 | 85 071 | 19·03 |
| **Mother's self-harm or mental disorder** |  |  |  |  |
| No | 405 791 | 87·27 | 390 196 | 87·29 |
| Yes | 59 217 | 12·73 | 56 807 | 12·71 |
| **Father's self-harm or mental disorder** |  |  |  |  |
| No | 408 250 | 87·79 | 392 348 | 87·77 |
| Yes | 56 758 | 12·21 | 54 655 | 12·23 |

* used in sensitivity analysis only

## Table S3: Number of cases and incidence rates of cohort members with a recorded self-harm episode during follow-up stratified by the covariates.

| **Covariate** | **Number of cases (total = 17 897)** | **Incidence rate (per 100 000 person-years)** |
| --- | --- | --- |
| **Sex** |  |  |
| Male | 8667 | 156·3 |
| Female | 9230 | 173·7 |
| **Birth year** |  |  |
| 1985 | 1396 | 126·6 |
| 1986 | 1347 | 132·0 |
| 1987 | 1330 | 139·6 |
| 1988 | 1408 | 149·0 |
| 1989 | 1371 | 154·2 |
| 1990 | 1418 | 165·2 |
| 1991 | 1289 | 163·0 |
| 1992 | 1276 | 173·2 |
| 1993 | 1166 | 173·3 |
| 1994 | 1135 | 183·0 |
| 1995 | 1053 | 191·9 |
| 1996 | 926 | 196·6 |
| 1997 | 833 | 205·7 |
| 1998 | 748 | 223·2 |
| 1999 | 689 | 239·5 |
| 2000 | 512 | 229·5 |
| **Area-level urbanicity** |  |  |
| Unknown | 721 | 784·4 |
| Rural | 3931 | 130·2 |
| Semi-urban | 3048 | 151·6 |
| Urban | 10197 | 177·7 |
| **Area-level morbidity** |  |  |
| Quintile 1 (lowest) | 3416 | 190·8 |
| Quintile 2 | 3617 | 170·9 |
| Quintile 3 | 3310 | 151·9 |
| Quintile 4 | 3732 | 159·9 |
| Quintile 5 (highest) | 3822 | 156·6 |
| **Area-level employment** |  |  |
| Quintile 1 (highest) | 3517 | 155·6 |
| Quintile 2 | 3497 | 161·7 |
| Quintile 3 | 3566 | 166·5 |
| Quintile 4 | 3444 | 163·1 |
| Quintile 5 (lowest) | 3873 | 177·4 |
| **Area-level education** |  |  |
| Quintile 1 (highest) | 3227 | 190·6 |
| Quintile 2 | 3319 | 182·8 |
| Quintile 3 | 3677 | 176·3 |
| Quintile 4 | 3862 | 158·2 |
| Quintile 5 (lowest) | 3812 | 135·0 |
| **Mother's education** |  |  |
| Primary | 3973 | 250·2 |
| Secondary | 8238 | 175·4 |
| Higher | 5686 | 124·3 |
| **Father's education** |  |  |
| Primary | 5893 | 225·0 |
| Secondary | 7889 | 167·9 |
| Higher | 4115 | 116·1 |
| **Mother's income** |  |  |
| Unknown | 435 | 297·5 |
| Quintile 1 (lowest) | 5174 | 221·4 |
| Quintile 2 | 3909 | 159·6 |
| Quintile 3 | 3263 | 147·3 |
| Quintile 4 | 2782 | 145·2 |
| Quintile 5 (highest) | 2334 | 129·9 |
| **Father's income** |  |  |
| Unknown | 1643 | 301·0 |
| Quintile 1 (lowest) | 4917 | 222·2 |
| Quintile 2 | 3474 | 151·0 |
| Quintile 3 | 3068 | 147·8 |
| Quintile 4 | 2642 | 137·2 |
| Quintile 5 (highest) | 2153 | 119·6 |
| **Mother's self-harm or mental disorder** |  |  |
| No | 14050 | 145·1 |
| Yes | 3847 | 326·7 |
| **Father's self-harm or mental disorder** |  |  |
| No | 14052 | 145·7 |
| Yes | 3845 | 316·8 |

## Table S4: Number of self-harm episodes by school grade.

| **School grade** | **Self-harm episodes** |
| --- | --- |
| 1 | 24 |
| 2 | 26 |
| 3 | 32 |
| 4 | 44 |
| 5 | 82 |
| 6 | 148 |
| 7 | 348 |
| 8 | 538 |
| 9 | 775 |

## Table S5: Associations between having ninth-grade schoolmates who had self-harmed and subsequent self-harm risk, using both stricter and broader classification of self-harm.

|  |  | **Schoolmates who had self-harmed (exposure)** | | | |
| --- | --- | --- | --- | --- | --- |
|  |  | **No (ref.)** |  | **Yes** | |
|  | **Follow-up period** | **n** |  | **n** | **HR (95% CI)** |
| **Strict classification** | Entire follow-up | 11 964 |  | 1950 | 1·06 (1·00 to 1·11) |
|  | Year 1 | 613 |  | 138 | 1·32 (1·09 to 1·59) |
|  | Year 2 | 809 |  | 159 | 1·08 (0·90 to 1·29) |
|  | Year 3 | 1126 |  | 232 | 1·16 (1·00 to 1·34) |
|  | Year 4 | 1342 |  | 272 | 1·14 (1·00 to 1·31) |
|  | Year 5 | 1300 |  | 247 | 1·14 (0·99 to 1·32) |
|  | Year 6 | 1185 |  | 186 | 1·01 (0·86 to 1·19) |
| **Broad classification** | Entire follow-up | 15 030 |  | 3979 | 1·06 (1·02 to 1·10) |
|  | Year 1 | 728 |  | 289 | 1·40 (1·21 to 1·61) |
|  | Year 2 | 939 |  | 291 | 1·03 (0·90 to 1·18) |
|  | Year 3 | 1332 |  | 436 | 1·09 (0·97 to 1·22) |
|  | Year 4 | 1561 |  | 526 | 1·15 (1·03 to 1·27) |
|  | Year 5 | 1594 |  | 471 | 1·10 (0·99 to 1·23) |
|  | Year 6 | 1403 |  | 392 | 1·10 (0·98 to 1·24) |

In the stricter classification, self-harm was defined using ICD-10 codes X60–X84, and in the broader classification, self-harm was defined using codes X60–X84, Y10–Y34, Y87.0, Y87.2, Z72.8, and Z91.5. Hazard ratios (HRs) with 95% confidence intervals shown for the entire follow-up and separately in one-year time windows for the first six years of follow-up. The n’s denote the number of cohort members who self-harmed during the follow-up period, stratified by the exposure. The Cox proportional hazards models were estimated independently for each time window and adjusted for sex, birth year, number of schoolmates in the same grade, area-level urbanicity, area-level morbidity, area-level education, area-level employment, parental education, parental income, and parental self-harm and mental health history, with a random intercept per school.

## Table S6: Associations between having ninth-grade schoolmates who had self-harmed and subsequent self-harm risk, stratified into two time periods: 2001–2008 and 2009–2016.

|  |  | **Schoolmates who had self-harmed (exposure)** | | | |
| --- | --- | --- | --- | --- | --- |
|  |  | **No (ref.)** |  | **Yes** | |
|  | **Follow-up period** | **n** |  | **n** | **HR (95% CI)** |
| **2001–2008** | Entire follow-up | 9277 |  | 1430 | 1·03 (0·97 to 1·09) |
|  | Year 1 | 312 |  | 65 | 1·46 (1·11 to 1·92) |
|  | Year 2 | 413 |  | 55 | 0·90 (0·67 to 1·19) |
|  | Year 3 | 568 |  | 111 | 1·27 (1·03 to 1·57) |
|  | Year 4 | 734 |  | 119 | 1·09 (0·90 to 1·33) |
|  | Year 5 | 814 |  | 143 | 1·19 (0·99 to 1·43) |
|  | Year 6 | 771 |  | 118 | 1·05 (0·86 to 1·28) |
| **2009–2016** | Entire follow-up | 5283 |  | 1907 | 1·09 (1·03 to 1·15) |
|  | Year 1 | 371 |  | 178 | 1·48 (1·23 to 1·78) |
|  | Year 2 | 487 |  | 185 | 1·06 (0·88 to 1·26) |
|  | Year 3 | 724 |  | 256 | 1·01 (0·87 to 1·17) |
|  | Year 4 | 791 |  | 322 | 1·15 (1·01 to 1·32) |
|  | Year 5 | 733 |  | 270 | 1·11 (0·96 to 1·28) |
|  | Year 6 | 607 |  | 212 | 1·12 (0·95 to 1·32) |

The n’s denote the number of cohort members who self-harmed during the follow-up period, stratified by the exposure. The Cox proportional hazards models were estimated independently for each time window and adjusted for sex, birth year, number of schoolmates in the same grade, area-level urbanicity, area-level morbidity, area-level education, area-level employment, parental education, parental income, and parental self-harm and mental health history, with a random intercept per school.

## Table S7: Descriptive statistics of the study population at the end of eighth (A) and seventh (B) grade.

| **A Eighth grade** | **Self-harm episode during grades 1–8** | | **No self-harm episode before end of grade 8** | |
| --- | --- | --- | --- | --- |
| **Characteristic** | **n** | **%** | **n** | **%** |
| **Sex** |  |  |  |  |
| Male | 544 | 38·50 | 464 340 | 51·00 |
| Female | 869 | 61·50 | 446 043 | 49·00 |
| **Birth year** |  |  |  |  |
| 1985 | 50 | 3·54 | 57 997 | 6·37 |
| 1986 | 54 | 3·82 | 56 575 | 6·21 |
| 1987 | 50 | 3·54 | 55 755 | 6·12 |
| 1988 | 58 | 4·10 | 58 617 | 6·44 |
| 1989 | 60 | 4·25 | 58 639 | 6·44 |
| 1990 | 55 | 3·89 | 60 473 | 6·64 |
| 1991 | 61 | 4·32 | 59 760 | 6·56 |
| 1992 | 53 | 3·75 | 60 016 | 6·59 |
| 1993 | 79 | 5·59 | 59 491 | 6·53 |
| 1994 | 105 | 7·43 | 60 012 | 6·59 |
| 1995 | 92 | 6·51 | 58 615 | 6·44 |
| 1996 | 122 | 8·63 | 56 177 | 6·17 |
| 1997 | 148 | 10·47 | 54 586 | 6·00 |
| 1998 | 132 | 9·34 | 51 997 | 5·71 |
| 1999 | 156 | 11·04 | 52 625 | 5·78 |
| 2000 | 138 | 9·77 | 49 048 | 5·39 |
| **Recorded self-harm episode** |  |  |  |  |
| X60-X84 Intentional self-harm | 822 | 58·17 | 14 557 | 1·60 |
| Y10-Y34 Event of undetermined intent | 479 | 33·90 | 4 757 | 0·52 |
| Y87.0 Sequelae of intentional self-harm | 4 | 0·28 | 64 | 0·01 |
| Y87.2 Sequelae of events of undetermined intent | 9 | 0·64 | 43 | 0·00 |
| *Z72.8 Other problems related to lifestyle, including self-damaging behavior | 155 | 10·97 | 1 473 | 0·16 |
| *Z91.5 Personal history of self-harm | 85 | 6·02 | 664 | 0·07 |
| Any of the above | 1 413 | 100·00 | 19 869 | 2·18 |
| **Same-grade schoolmates with a recorded self-harm episode** |  |  |  |  |
| No | 151 | 10·69 | 798 191 | 87·68 |
| Yes | 1 262 | 89·31 | 112 192 | 12·32 |
| **Number of schoolmates in the same grade** | 109·45 | 48·12 | 112·50 | 47·85 |
| **Area-level urbanicity** |  |  |  |  |
| Unknown | 138 | 9·77 | 8 916 | 0·98 |
| Rural | 286 | 20·24 | 255 019 | 28·01 |
| Semi-urban | 217 | 15·36 | 172 622 | 18·96 |
| Urban | 772 | 54·64 | 473 826 | 52·05 |
| **Area-level morbidity** |  |  |  |  |
| Quintile 1 (lowest) | 324 | 22·93 | 183 714 | 20·18 |
| Quintile 2 | 325 | 23·00 | 182 521 | 20·05 |
| Quintile 3 | 261 | 18·47 | 182 178 | 20·01 |
| Quintile 4 | 292 | 20·67 | 180 830 | 19·86 |
| Quintile 5 (highest) | 211 | 14·93 | 181 140 | 19·90 |
| **Area-level employment** |  |  |  |  |
| Quintile 1 (highest) | 242 | 17·13 | 182 119 | 20·00 |
| Quintile 2 | 281 | 19·89 | 182 249 | 20·02 |
| Quintile 3 | 271 | 19·18 | 181 992 | 19·99 |
| Quintile 4 | 305 | 21·59 | 181 987 | 19·99 |
| Quintile 5 (lowest) | 314 | 22·22 | 182 036 | 20·00 |
| **Area-level education** |  |  |  |  |
| Quintile 1 (highest) | 380 | 26·89 | 182 008 | 19·99 |
| Quintile 2 | 344 | 24·35 | 182 032 | 20·00 |
| Quintile 3 | 286 | 20·24 | 182 035 | 20·00 |
| Quintile 4 | 224 | 15·85 | 182 107 | 20·00 |
| Quintile 5 (lowest) | 179 | 12·67 | 182 201 | 20·01 |
| **Mother’s education** |  |  |  |  |
| Primary | 280 | 19·82 | 124 812 | 13·71 |
| Secondary | 643 | 45·51 | 388 723 | 42·70 |
| Higher | 490 | 34·68 | 396 848 | 43·59 |
| **Father’s education** |  |  |  |  |
| Primary | 443 | 31·35 | 208 593 | 22·91 |
| Secondary | 619 | 43·81 | 398 709 | 43·80 |
| Higher | 351 | 24·84 | 303 081 | 33·29 |
| **Mother’s income** |  |  |  |  |
| Unknown | 35 | 2·48 | 11 033 | 1·21 |
| Quintile 1 (lowest) | 345 | 24·42 | 179 789 | 19·75 |
| Quintile 2 | 243 | 17·20 | 179 897 | 19·76 |
| Quintile 3 | 253 | 17·91 | 179 902 | 19·76 |
| Quintile 4 | 270 | 19·11 | 179 879 | 19·76 |
| Quintile 5 (highest) | 267 | 18·90 | 179 883 | 19·76 |
| **Father’s income** |  |  |  |  |
| Unknown | 150 | 10·62 | 42 928 | 4·72 |
| Quintile 1 (lowest) | 358 | 25·34 | 173 369 | 19·04 |
| Quintile 2 | 229 | 16·21 | 173 526 | 19·06 |
| Quintile 3 | 221 | 15·64 | 173 529 | 19·06 |
| Quintile 4 | 231 | 16·35 | 173 508 | 19·06 |
| Quintile 5 (highest) | 224 | 15·85 | 173 523 | 19·06 |
| **Mother’s self-harm or mental disorder** |  |  |  |  |
| No | 998 | 70·63 | 805 545 | 88·48 |
| Yes | 415 | 29·37 | 104 838 | 11·52 |
| **Father’s self-harm or mental disorder** |  |  |  |  |
| No | 1 081 | 76·50 | 806 588 | 88·60 |
| Yes | 332 | 23·50 | 103 795 | 11·40 |

| **B Seventh grade** | **Self-harm episode during grades 1–7** | | **No self-harm episode before end of grade 7** | |
| --- | --- | --- | --- | --- |
| **Characteristic** | **n** | **%** | **n** | **%** |
| **Sex** |  |  |  |  |
| Male | 373 | 45·94 | 464 511 | 50·99 |
| Female | 439 | 54·06 | 446 473 | 49·01 |
| **Birth year** |  |  |  |  |
| 1985 | 26 | 3·20 | 58 021 | 6·37 |
| 1986 | 33 | 4·06 | 56 596 | 6·21 |
| 1987 | 27 | 3·33 | 55 778 | 6·12 |
| 1988 | 30 | 3·69 | 58 645 | 6·44 |
| 1989 | 36 | 4·43 | 58 663 | 6·44 |
| 1990 | 37 | 4·56 | 60 491 | 6·64 |
| 1991 | 32 | 3·94 | 59 789 | 6·56 |
| 1992 | 34 | 4·19 | 60 035 | 6·59 |
| 1993 | 40 | 4·93 | 59 530 | 6·53 |
| 1994 | 62 | 7·64 | 60 055 | 6·59 |
| 1995 | 60 | 7·39 | 58 647 | 6·44 |
| 1996 | 74 | 9·11 | 56 225 | 6·17 |
| 1997 | 73 | 8·99 | 54 661 | 6·00 |
| 1998 | 83 | 10·22 | 52 046 | 5·71 |
| 1999 | 93 | 11·45 | 52 688 | 5·78 |
| 2000 | 72 | 8·87 | 49 114 | 5·39 |
| **Recorded self-harm episode** |  |  |  |  |
| X60-X84 Intentional self-harm | 390 | 48·03 | 14 989 | 1·65 |
| Y10-Y34 Event of undetermined intent | 336 | 41·38 | 4 900 | 0·54 |
| Y87.0 Sequelae of intentional self-harm | na | na | 66 | 0·01 |
| Y87.2 Sequelae of events of undetermined intent | 4 | 0·49 | 48 | 0·01 |
| *Z72.8 Other problems related to lifestyle, including self-damaging behavior | 98 | 12·07 | 1 530 | 0·17 |
| *Z91.5 Personal history of self-harm | 45 | 5·54 | 704 | 0·08 |
| Any of the above | na | 100·00 | 20 470 | 2·25 |
| **Same-grade schoolmates with a recorded self-harm episode** |  |  |  |  |
| No | 105 | 12·93 | 844 742 | 92·73 |
| Yes | 707 | 87·07 | 66 242 | 7·27 |
| **Number of schoolmates in the same grade** | 108·36 | 48·14 | 112·49 | 47·85 |
| **Area-level urbanicity** |  |  |  |  |
| Unknown | 72 | 8·87 | 8 054 | 0·88 |
| Rural | 175 | 21·55 | 263 430 | 28·92 |
| Semi-urban | 140 | 17·24 | 174 149 | 19·12 |
| Urban | 425 | 52·34 | 465 351 | 51·08 |
| **Area-level morbidity** |  |  |  |  |
| Quintile 1 (lowest) | 181 | 22·29 | 182 460 | 20·03 |
| Quintile 2 | 172 | 21·18 | 181 997 | 19·98 |
| Quintile 3 | 163 | 20·07 | 182 512 | 20·03 |
| Quintile 4 | 167 | 20·57 | 183 377 | 20·13 |
| Quintile 5 (highest) | 129 | 15·89 | 180 638 | 19·83 |
| **Area-level employment** |  |  |  |  |
| Quintile 1 (highest) | 147 | 18·10 | 182 276 | 20·01 |
| Quintile 2 | 157 | 19·33 | 182 132 | 19·99 |
| Quintile 3 | 170 | 20·94 | 182 199 | 20·00 |
| Quintile 4 | 158 | 19·46 | 182 237 | 20·00 |
| Quintile 5 (lowest) | 180 | 22·17 | 182 140 | 19·99 |
| **Area-level education** |  |  |  |  |
| Quintile 1 (highest) | 221 | 27·22 | 182 162 | 20·00 |
| Quintile 2 | 212 | 26·11 | 182 113 | 19·99 |
| Quintile 3 | 161 | 19·83 | 182 204 | 20·00 |
| Quintile 4 | 121 | 14·90 | 182 232 | 20·00 |
| Quintile 5 (lowest) | 97 | 11·95 | 182 273 | 20·01 |
| **Mother’s education** |  |  |  |  |
| Primary | 176 | 21·67 | 127 204 | 13·96 |
| Secondary | 362 | 44·58 | 388 765 | 42·68 |
| Higher | 274 | 33·74 | 395 015 | 43·36 |
| **Father’s education** |  |  |  |  |
| Primary | 259 | 31·90 | 208 479 | 22·89 |
| Secondary | 348 | 42·86 | 399 440 | 43·85 |
| Higher | 205 | 25·25 | 303 065 | 33·27 |
| **Mother’s income** |  |  |  |  |
| Unknown | 20 | 2·46 | 10 119 | 1·11 |
| Quintile 1 (lowest) | 194 | 23·89 | 180 122 | 19·77 |
| Quintile 2 | 151 | 18·60 | 180 178 | 19·78 |
| Quintile 3 | 130 | 16·01 | 180 210 | 19·78 |
| Quintile 4 | 164 | 20·20 | 180 168 | 19·78 |
| Quintile 5 (highest) | 153 | 18·84 | 180 187 | 19·78 |
| **Father’s income** |  |  |  |  |
| Unknown | 93 | 11·45 | 40 397 | 4·43 |
| Quintile 1 (lowest) | 206 | 25·37 | 174 029 | 19·10 |
| Quintile 2 | 127 | 15·64 | 174 149 | 19·12 |
| Quintile 3 | 122 | 15·02 | 174 155 | 19·12 |
| Quintile 4 | 137 | 16·87 | 174 117 | 19·11 |
| Quintile 5 (highest) | 127 | 15·64 | 174 137 | 19·12 |
| **Mother’s self-harm or mental disorder** |  |  |  |  |
| No | 596 | 73·40 | 815 914 | 89·56 |
| Yes | 216 | 26·60 | 95 070 | 10·44 |
| **Father’s self-harm or mental disorder** |  |  |  |  |
| No | 632 | 77·83 | 814 013 | 89·36 |
| Yes | 180 | 22·17 | 96 971 | 10·64 |

* Included in sensitivity analysis only.

Note: Data with fewer than three observations are omitted due to data protection regulations.

## Table S8: Associations between having same-grade schoolmates who self-harmed and the risk of subsequent self-harm in lower secondary school, with follow-up starting at the end of each respective grade.

|  |  | **Schoolmates who had self-harmed (exposure)** | | | |
| --- | --- | --- | --- | --- | --- |
|  |  | **No (ref.)** |  | **Yes** | |
|  | **Follow-up period** | **n** |  | **n** | **HR (95% CI)** |
| **seventh grade** | Entire follow-up | 17 838 |  | 1400 | 1·04 (0·98 to 1·10) |
|  | Year 1 | 488 |  | 47 | 0·98 (0·72 to 1·33) |
|  | Year 2 | 689 |  | 82 | 1·26 (1·00 to 1·60) |
|  | Year 3 | 845 |  | 88 | 1·25 (1·00 to 1·57) |
|  | Year 4 | 1036 |  | 109 | 1·16 (0·94 to 1·43) |
|  | Year 5 | 1524 |  | 141 | 1·03 (0·86 to 1·23) |
|  | Year 6 | 1800 |  | 170 | 1·09 (0·92 to 1·28) |
| **eighth grade** | Entire follow-up | 17 620 |  | 1063 | 1·07 (1·00 to 1·14) |
|  | Year 1 | 710 |  | 58 | 1·13 (0·86 to 1·49) |
|  | Year 2 | 859 |  | 73 | 1·36 (1·07 to 1·74) |
|  | Year 3 | 1080 |  | 62 | 0·83 (0·64 to 1·08) |
|  | Year 4 | 1554 |  | 108 | 1·02 (0·84 to 1·25) |
|  | Year 5 | 1814 |  | 153 | 1·29 (1·09 to 1·53) |
|  | Year 6 | 1835 |  | 128 | 1·20 (1·00 to 1·45) |
| **ninth grade** | Entire follow-up | 16 425 |  | 1472 | 1·06 (1·00 to 1·12) |
|  | Year 1 | 812 |  | 114 | 1·49 (1·21 to 1·82) |
|  | Year 2 | 1029 |  | 111 | 1·10 (0·90 to 1·34) |
|  | Year 3 | 1493 |  | 166 | 1·14 (0·96 to 1·34) |
|  | Year 4 | 1778 |  | 188 | 1·09 (0·93 to 1·27) |
|  | Year 5 | 1782 |  | 178 | 1·15 (0·98 to 1·34) |
|  | Year 6 | 1556 |  | 152 | 1·14 (0·96 to 1·36) |

The n’s denote the number of cohort members who self-harmed during the follow-up period, stratified by the exposure· The Cox proportional hazards models were estimated independently for each time window and adjusted for sex, birth year, number of schoolmates in the same grade, area-level urbanicity, area-level morbidity, area-level education, area-level employment, parental education, parental income, and parental self-harm and mental health history, with a random intercept per school.

Figure S1: Associations between having ninth-grade schoolmates who had self-harmed and subsequent self-harm risk, using both stricter and broader classification of self-harm. In the stricter classification, self-harm was defined using ICD-10 codes X60–X84, and in the broader classification, self-harm was defined using codes X60–X84, Y10–Y34, Y87.0, Y87.2, Z72.8, and Z91.5. The associations are shown in one-year time windows for the first six years of follow-up. The error bars represent the 95% confidence interval. The Cox proportional hazards models were estimated independently for each time window and adjusted for sex, birth year, number of schoolmates in the same grade, area-level urbanicity, area-level morbidity, area-level education, area-level employment, parental education, parental income, and parental self-harm and mental health history, with a random intercept per school.

Figure S2: Associations between having ninth-grade schoolmates who had self-harmed and subsequent self-harm risk, stratified into two time periods: 2001–2008 and 2009–2016. The associations are shown in one-year time windows for the first six years of follow-up. The error bars represent the 95% confidence interval. The Cox proportional hazards models were estimated independently for each time window and adjusted for sex, birth year, number of schoolmates in the same grade, area-level urbanicity, area-level morbidity, area-level education, area-level employment, parental education, parental income, and parental self-harm and mental health history, with a random intercept per school. The arrow indicates that the confidence interval extends outside the range of the graph.

Figure S3: Associations between having same-grade schoolmates who self-harmed and the risk of subsequent self-harm in lower secondary school, with follow-up starting at the end of each grade. The associations are shown in one-year time windows for the first six years of follow-up. The error bars represent the 95% confidence interval. The Cox proportional hazards models were estimated independently for each time window and adjusted for sex, birth year, number of schoolmates in the same grade, area-level urbanicity, area-level morbidity, area-level education, area-level employment, parental education, parental income, and parental self-harm and mental health history, with a random intercept per school.
